# Metallization and Electrical Transport Behaviors of GaSb under High-Pressure

**DOI:** 10.1038/s41598-017-02592-5

**Published:** 2017-06-01

**Authors:** Guozhao Zhang, Baojia Wu, Jia Wang, Haiwa Zhang, Hao Liu, Junkai Zhang, Cailong Liu, Guangrui Gu, Lianhua Tian, Yanzhang Ma, Chunxiao Gao

**Affiliations:** 1 Department of Physics, College of Science, Yanbian University, Yanji Jilin, 133002 China; 2 State Key Lab for Superhard Materials, Institute of Atomic and Molecular Physics and Department of Materials Science, Jilin University, Changchun, 130012 China; 3 Key Laboratory of Functional Materials Physics and Chemistry of the Ministry of Education, Jilin Normal University, Siping, 136000 China; 4 Department of Mechanical Engineering, Texas Tech University, Lubbock, Texas 79409 USA

## Abstract

The high-pressure metallization and electrical transport behaviors of GaSb were systematically investigated using *in situ* temperature-dependent electrical resistivity measurements, Hall effect measurements, transmission electron microscopy analysis, and first-principles calculations. The temperature-dependent resistivity measurements revealed pressure-induced metallization of GaSb at approximately 7.0 GPa, which corresponds to a structural phase transition from *F-43m* to *Imma*. In addition, the activation energies for the conductivity and Hall effect measurements indicated that GaSb undergoes a carrier-type inversion (p-type to n-type) at approximately 4.5 GPa before metallization. The first-principles calculations also revealed that GaSb undergoes a phase transition from *F-43m* to *Imma* at 7.0 GPa and explained the carrier-type inversion at approximately 4.5 GPa. Finally, transmission electron microscopy analysis revealed the effect of the interface on the electrical transport behavior of a small-resistance GaSb sample and explained the discontinuous change of resistivity after metallization. Under high pressure, GaSb undergoes grain refinement, the number of interfaces increases, and carrier transport becomes more difficult, increasing the electrical resistivity.

## Introduction

Pressure-induced metallization has been observed in many materials, including VO_2_^1^, MgO^2^, and SiH_4_^3^. However, different materials are characterized by different pressure-induced metallization processes. For example, under high pressure, SnO^4^ undergoes p-type to n-type metallization, whereas Mg_2_Ge^5^ and Ag_2_S^6^ undergo n-type to p-type metallization. Understanding the pressure-induced metallization process of materials is critical for analysis of their conductivity mechanisms as well as for their applications.

The III–V semiconductor material GaSb has attracted considerable attention because of its value in applications such as high-speed electronics and infrared equipment^7–11^. For example, the conversion efficiency of a GaAs/GaSb tandem solar cell with GaSb as the substrate can reach over 35%^12^. Under ambient conditions, the carrier concentration of a typical tetragonal p-type semiconductor composed of pure GaSb is 1 × 10^17^ *cm* ^−3^, with a corresponding band gap of 0.72 eV and lattice parameter of 0.6095 Å. Research on GaSb under high-pressure conditions is on-going as experimental technologies develop, in particular, further investigation of the pressure-induced phase transitions of GaSb is needed. The study of GaSb metallization can be traced back to 1962 when Minomura *et al*. observed a sudden decrease of the resistance of GaSb at 8.0–10.0 GPa in high-pressure resistance experiments; the authors considered the second phase to most likely be a metallic state^13^. X-ray powder diffraction (XRD) studies first characterized the structure as a site-ordered metallic β-Sn-like structure similar to that for Ge and Si at 7 GPa^14, 15^. Later, angle-dispersive X-ray (ADXRD) powder diffraction experiments were performed in a diamond anvil cell (DAC) with a 4:1 mixture of methanol:ethanol as the pressure-transmitting medium. A disordered orthorhombic structure with space group *Imma* was observed rather than the previously reported β-Sn structure; however, the authors did not report whether metallization had occurred^16^. In 1999, Mezouar *et al*. performed ADXRD experiments in a DAC using different pressure mediums to determine the effect of pressure on the high-pressure phase of GaSb and observed that the β-Sn structure could only be obtained under hydrostatic conditions^17^. Thus, the metal phase transition of GaSb has been inferred from high-pressure XRD and resistance measurements; however, as GaSb undergoes a phase transition from *F*-43*m* to *Imma* at 7.0 GPa under non-hydrostatic conditions, it remains unknown if the *Imma* phase is a metallic state. Furthermore, XRD measurements cannot effectively reflect the electronic structure phase transition of the material, which demonstrates the need to conduct electrical property studies under pressure.

The electrical conductivity of materials under pressure is strongly affected by interfaces^18, 19^. Therefore, it is necessary to study both the electrical properties and the effect of interfaces on these properties in samples under high pressure. Zhang *et al*. proposed a method to analyze the effect of interfaces on the electrical properties of Zn_2_SnO_4_ by combining AC impedance and transmission electron microscopy (TEM) analyses in 2015^20^. However, the AC impedance analyzer used (1296/1260, Solartron Analytical) could not detect electrical properties in the low-frequency region for a low-resistance sample. To date, no effective method has been proposed for analyzing the electrical properties and interface effect of a low-resistance sample under pressure.

In this study, the metallization of GaSb was systemically investigated using *in situ ρ* measurements, Hall effect measurements, and first-principles calculations under high pressure. The results provide sufficient evidence for the metallization of the material at 7.0 GPa. Before metallization, GaSb underwent a carrier-type inversion at approximately 4.5 GPa, which was caused by band gap changes with pressure, inducing a change in the impurity level from the acceptor to the donor level. We explain the electronic property change of the sample after metallization through examining the crystal interface of sample powders by high-resolution TEM (HRTEM) after pressure relief.

## Experimental Results and Discussion

**Figure 1:**
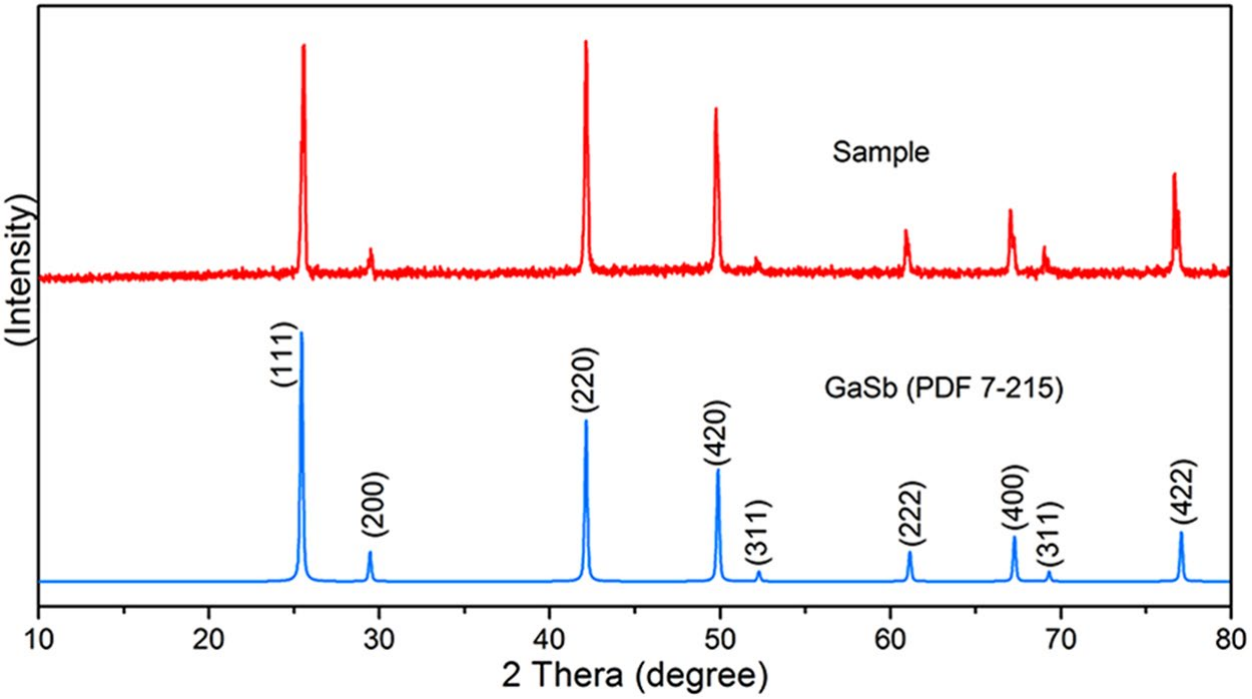
The comparison of XRD spectra of sample after mechanical grinding and PDF card No. 7-215.

GaSb (99.9%) was purchased from Alpha Company (USA), and the required powders were obtained by mechanical grinding. We conducted XRD measurements of the mechanically ground sample powders and compared the results with those of JCPDS No. 7–215, as shown in Fig. 1. The approximate particle size of the sample by was calculated by the Scherrer equation^21, 22^:

1 $$D=K\gamma /B\,{\rm{\cos }}\,\theta $$

where *D* is the mean particle size of the sample powders; *K* is a dimensionless shape factor, which is defined as 0.89 here; *γ* is the X-ray wavelength of 0.154056 nm; *B* is the line broadening at half the maximum intensity; and *θ* is the Bragg angle. The calculated result was 35.6 nm.

### *In Situ* High-pressure Electrical Resistivity Measurements

**Figure 2:**
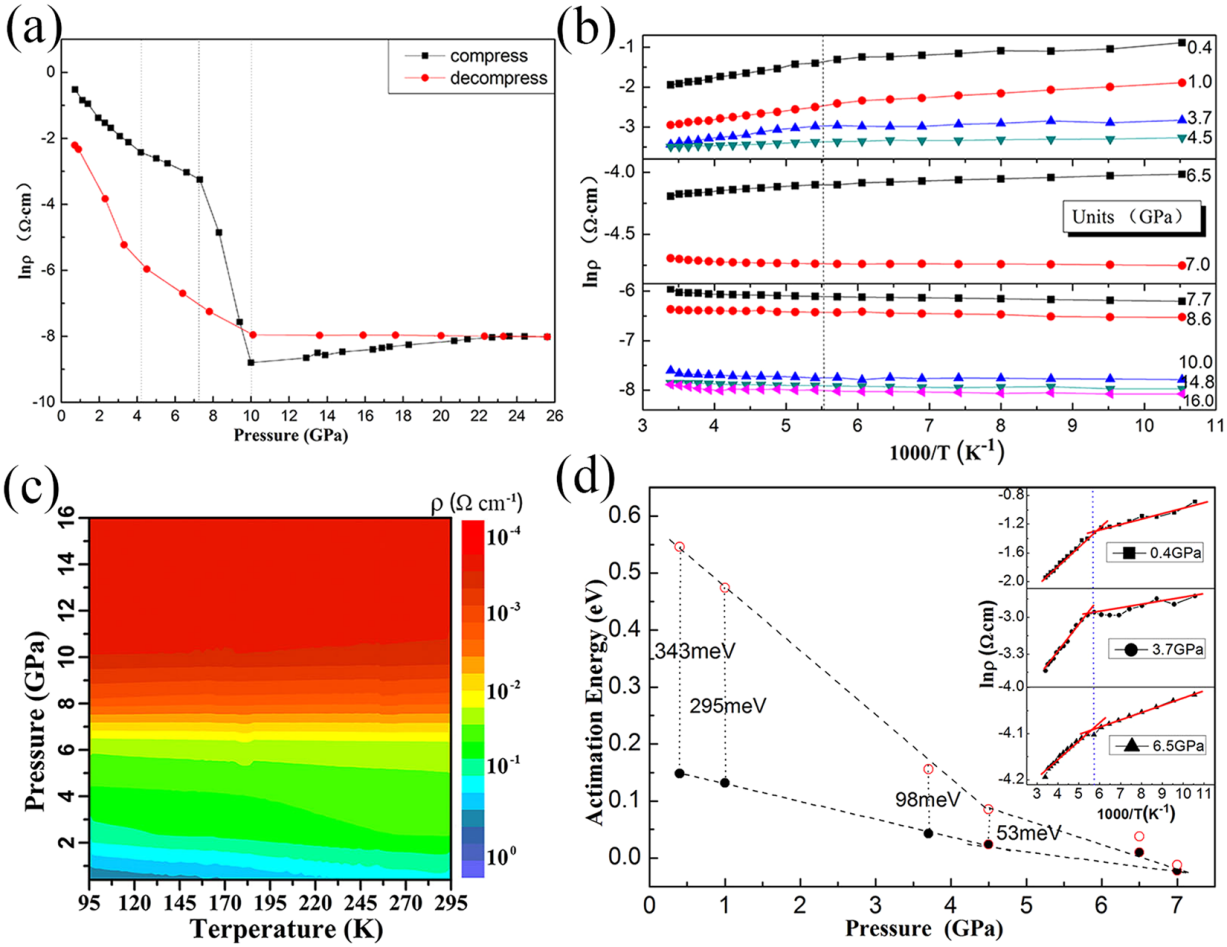
(**a**) Pressure dependence of electrical resistivity of GaSb at room temperature, dashed line positions indicate discontinuous pressure; (**b**) Temperature-resistivity curves at different pressure; (**c**) Temperature-pressure-resistivity contour map; (**d**) Pressure dependence of activation energy of GaSb in two different temperature regions: between 185 K and room temperature (solid circle), between 70 and 185 K (open circle). The inset shows the plots of ln *ρ vs* 1000/T at 0.4, 3.7, and 6.5 GPa.

As observed in Fig. 2(a), *ρ* decreased with increasing pressure below 10.0 GPa, with a sharp decline in the range of 7.0–10.0 GPa. Above 10.0 GPa, *ρ* slowly increased until 13.0 GPa and finally plateaued. Three regions with varying reduction rates for ln*ρ* were observed below 10.0 GPa: from ambient pressure to 4.5, 4.5 to 7.0 GPa, and 7.0 to 10.0 GPa, the reduction rates were 0.62, 0.26, and 2.22 *Ω·cm*/GPa, respectively. Therefore, we suspected that metallization of GaSb occurred between 7.0 and 10.0 GPa. After pressure relief, *ρ* returned to its original order of magnitude, indicating that the change of the sample was reversible under high pressure, which is consistent with previous research^16^.

To verify whether GaSb undergoes pressure-induced metallization, we conducted high-pressure variable-temperature *ρ* measurements. The experimental results could be fitted using an Arrhenius equation, as shown in Fig. 2(b). Below 7.0 GPa, *ρ* decreased with increasing temperature, and the material exhibited semiconductor conductivity characteristics; above 7 GPa, *ρ* increased with increasing temperature, and the material exhibited clear metal conductivity. These results demonstrate that GaSb underwent a typical phase transition from semiconductor to metal near 7.0 GPa. Above 10.0 GPa, *ρ* remained almost constant with increasing pressure (Fig. 2(c)).

To further explore the evolution process and intrinsic conductivity mechanism of GaSb metallization, the change in the activation energy of GaSb with increasing pressure was studied. The activation energy for carrier conductivity of GaSb was determined based on the change of *ρ* with temperature:

2 $$\rho ={\rho }_{0}\mathrm{Exp}({E}_{{\rm{I}}}/2{k}_{{\rm{B}}}T)$$

where *ρ*_0_ is a constant determined by both the carrier mobility *μ* and the effective mass of the carrier, *E*_I_ is the activation energy for conductivity, *k*_*B*_ is the Boltzmann constant, and *T* is the temperature.

The activation energy is generally temperature independent; however, we observed that the changes in *ρ* differed with increasing temperature when determining the activation energy of GaSb through fitting. Under different pressures, ln *ρ vs*. 1000/*T* could be linearly fitted in two sections: the changes in *ρ* with increasing temperature from 95 to 185 K were gentler than those above 185 K. In other words, the activation energy in the high-temperature range from 185 K to room temperature was greater than that in the low-temperature range from 95 to 185 K under the same pressure. This result was observed because dislocations appeared at the interface of the GaSb sample powder after mechanical grinding^23^, resulting in impurity energy levels in the band gap. The carriers participating in the conductivity could be excited to the low-impurity energy level in the low-temperature range from 95 to 185 K. The activation energy in the low-temperature range of 95 to 185 K was relatively low. With increasing temperature, the excited carriers became saturated and were not easily further excited to a higher impurity level.

Figure 2(d) clearly shows that the activation energies in the two temperature ranges were both reduced with increasing pressure. This finding reflects the effect of pressure on the charge-transfer energy barrier. At 7.0 GPa, *E*_I_ was close to 0; the typical semiconductor behavior of GaSb and accompanying energy barrier disappeared. This result indicates that there were no barriers to overcome in the carrier migration process. In other words, at this time, GaSb exhibited electronic conductivity, which is characteristic of metal conduction behavior. In addition, the negative slope indicates that the transport of carriers became easier with increasing pressure, and that the change in the activation energy for the conductivity of GaSb in different temperature ranges above and below 4.5 GPa showed two different trends. The reduction rates of the activation energy in the two temperature ranges below 4.5 GPa were smaller than those above 4.5 GPa, which corresponds to the inflection point of the high-pressure *in situ ρ* measurements at 4.5 GPa. However, no structural transformation was observed before metallization in previous XRD studies; therefore, we suspect that GaSb underwent an electronic structural phase transition from a p-type to an n-type semiconductor.

### *In Situ* Hall Effect Measurements Under High Pressure

**Figure 3:**
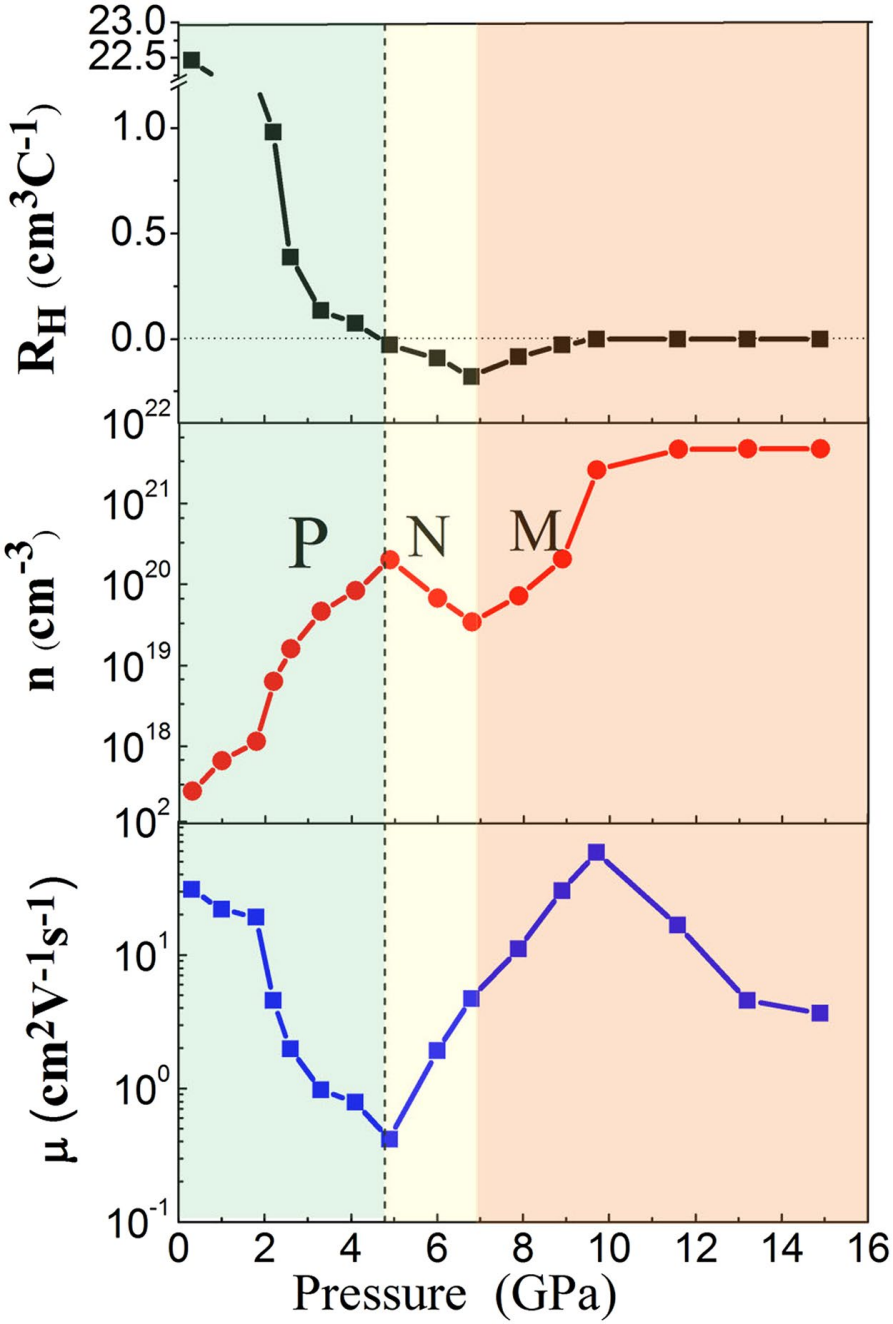
Pressure dependence of Hall coefficient, carrier concentration, and mobility of GaSb at room temperature. P, N, M represent P-type semiconductor, N-type semiconductor and Metallization, respectively. The vertical dashed line indicates the carrier-type inversion.

Figure 3 presents the Hall effect measurements of GaSb for a magnetic field of 1.0 T. Under ambient pressure, the Hall coefficient *R*_H_, carrier concentration *n*, and *μ* were 22.47 *cm* ^*3*^ *C* ^−*1*^, 1.00 × 10^17^ *cm* ^−*3*^, and 30.81 *cm* ^*2*^ *V* ^−*1*^ *s* ^−*1*^, respectively. Before metallization, *R*_H_ changed from positive to negative at approximately 4.5 GPa, which indicates that the sample underwent carrier-type inversion, changing from a p-type to an n-type semiconductor. Below 4.5 GPa, *n* decreased and *μ* increased with increasing pressure as observed in Fig. 3; therefore, the reduction of *μ* caused the reduction of ln *ρ*, and the reduction rate of ln *ρ* changed from 0.62 to 0.26 *Ω·cm*/GPa. At 4.5 GPa, the numbers of electrons and holes added were equal. Above 4.5 GPa, the number of electrons increased with increasing pressure, and the number of holes remained constant; however, the creation rate of electrons was less than the annihilation rate of electrons and holes. Therefore, *n* decreased with increasing pressure from 4.5 to 7.0 GPa. In the metallization process, the creation rate of electrons was greater than the annihilation rate of electrons and holes above 7.0 GPa; in addition, *n* and *μ* increased with increasing pressure and ln *ρ* decreased sharply at a rate of 2.22 *Ω·cm*/GPa.

After metallization, *R*_H_ was unchanged and close to 0, and *n* remained approximately 4.83 cm^−3^ and showed no change with increasing pressure. At this time, the excited carriers approached saturation; however, *μ* decreased with increasing pressure^4^. It could be assumed that the increasing *ρ* of the sample was caused by the reduction of *μ*. Above 13 GPa, *μ* remained as 3.74 *cm* ^*2*^ *V* ^−*1*^ *s* ^−*1*^ and showed no change with increasing pressure, and *ρ* showed no change.

### HRTEM Analysis after Decompression

The high-pressure *in situ ρ* measurements indicated that *ρ* of GaSb remained unchanged after a slight increase above 10.0 GPa. The high-pressure *in situ* Hall effect measurements also did not reveal an electronic phase transition; however, *μ* decreased with increasing pressure. No structural phase transition was observed in GaSb near this pressure in previous XRD experiments. The reduction of *μ* was thought to be associated with crystal refinement or breaking. Crystal breaking or refinement generally implies the generation of a new interface, which will result in physical property changes. To analyze this effect, HRTEM characterization was performed on the GaSb powders after relieving pressures of 5.0, 10.0, and 25.0 GPa.

**Figure 4:**
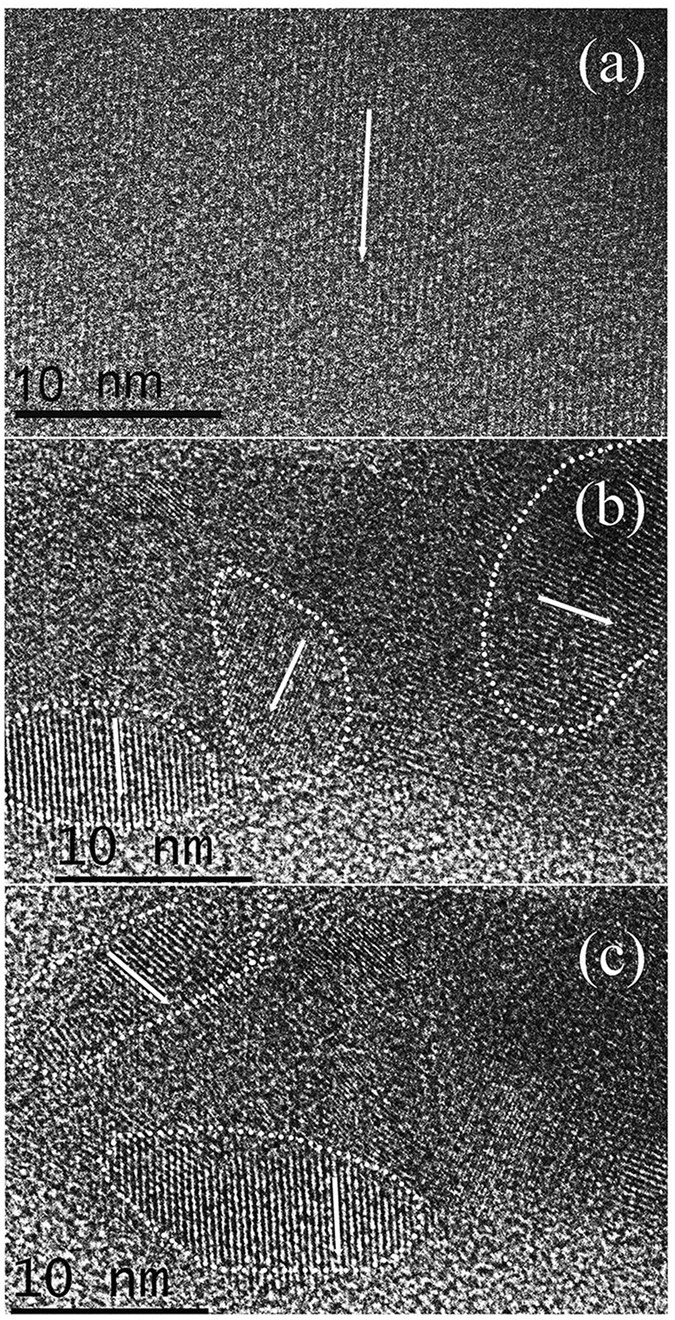
TEM images of the GaSb samples after decompression from different pressures. (**a**) 5.0 GPa; (**b**) 10.0 GPa; (**c**) 25.0 GPa.

The characterization results are presented in Fig. 4. Comparison of Fig. 4(a–c) reveals that the sample maintained the long-range order characteristic of a single crystal over a large area after relief of 5.0 GPa. However, after relief of 10.0 GPa, the sample was partially crushed, and the lattice direction was disordered. After relief of 25.0 GPa, the sample was similar to that after relief of 10.0 GPa without further refinement. Therefore, the same refinement produced many new interfaces, and it was difficult for the carriers to move in the crystal. Thus, above 10.0 GPa, *μ* decreased and *ρ* increased slightly. This finding may be caused by non-hydrostatic pressures in the DAC.

## Theoretical Calculation Results

**Figure 5:**
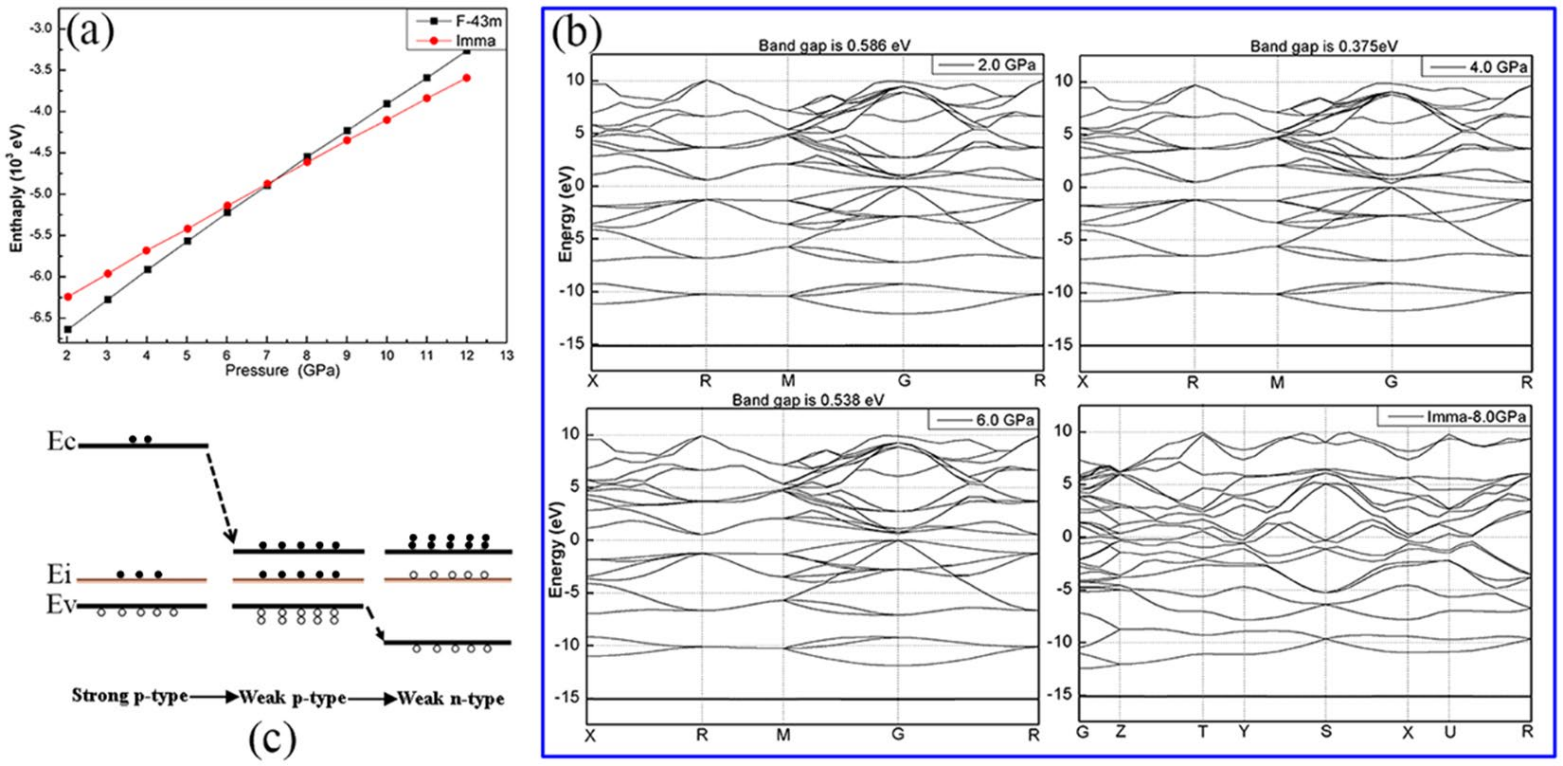
(**a**) The change enthalpy vs. pressure relationships of the *F-43m* and *Imma* phases of GaSb; (**b**) Calculated band structure under different pressure; (**c**) A sketch for the changing of band structure in *F-43m* phase; Ec, Ev, and Ei represent the conduction-band minimum, valence-band maximum, and impurity level; the soild and open circles repersent the electrons and holes, respectively.

The theoretical calculation results are presented in Fig. 5. The enthalpy of the *F-43m* and *Imma* phases intersect at 7.1 GPa, which indicates that the *Imma* phase of GaSb is more stable than *F-43m* above 7.1 GPa. The band structure calculation results indicate that the *F-43m* structure of GaSb is a direct-band-gap semiconductor. For the *Imma* phase, the band gap across the Fermi level indicates that the *Imma* phase is metallic. These theoretical calculation results agree with our experimental data, confirming the metallization of GaSb near 7.0 GPa.

Before metallization, the band gaps of GaSb were 0.586, 0.375, and 0.538 eV at 2.0, 4.0, and 6.0 GPa, respectively. Thus, the band gap first decreased and then increased with increasing pressure, corresponding to the carrier-type inversion before metallization. A low concentration of impurities and defects is present in commercially obtained GaSb; therefore, impurity levels are present in the band gap. At ambient pressure, the impurity level is close to the valence band, and electrons can move more easily from the valence band to the impurity levels. Therefore, the impurity level acts as an acceptor level, and the conductivity depends mostly on holes; thus, GaSb is a strong p-type semiconductor. With increasing pressure, the conduction band approaches the impurity levels, and the number of electrons increases in the conduction band; therefore, GaSb gradually becomes a weak p-type semiconductor. When the pressure is increased further, the valence band moves away from the impurity levels and electrons more easily move from the impurity levels to the conduction band. Therefore, the impurity level acts as a donor level, the conductivity mostly relies on electrons, and GaSb becomes an n-type semiconductor, as observed in Fig. 5(c).

## Conclusion

Using high-pressure *in situ ρ* measurements, we confirmed that the metallization process of GaSb starts at 7.0 GPa and observed two anomalous changes of *ρ* at 4.5 and 10.0 GPa before and after metallization, respectively. High-pressure Hall measurements revealed that GaSb undergoes a carrier-type inversion from a p-type to an n-type semiconductor as a pre-process for metallization at 4.5 GPa. These effects were explained by theoretical calculations, which revealed that the changes of the band structure with increasing pressure transform the impurity levels from acceptor to donor levels. In addition, TEM analysis and Hall effect measurements elucidated the effect of the interface on the electrical transport behavior of small-resistance GaSb samples under high pressure and explained the discontinuous change of *ρ* after metallization. GaSb undergoes grain refinement under high pressure; thus, the number of interfaces increases, making carrier transport more difficult and decreasing *μ* and increasing *ρ*.

## Methods

The high-pressure device was a Mao–Bell^24^ DAC, with an anvil face of 300 μm. The laser drilling technique was used to drill a hole with a diameter of 100 μm in a non-magnetic Re gasket with a thickness of 50 μm as the sample cavity. The hole was filled with a mixture of alumina powder and epoxy resin as the insulator. Because of the possibility of anvil deformation, the thickness of the sample under high pressure was measured with a micrometer with an accuracy of up to 0.5 μm, and the pressure was standardized by using the ruby fluorescence spectra method^25, 26^. To avoid the introduction of impurities during the measurements and to ensure good electrical contact for the electronic parameter measurements, a pressure transmission medium was not used in the experiments. The high-pressure *ρ* and Hall effect measurements were performed in the DAC integrated microcircuit using the van der Pauw method^27, 28^.

In the *ρ* and Hall effect measurements, source meters (2400 and 2700, Keithley) were used to provide the current and measure the voltage, respectively. All the instruments were connected to the computer using an interface adapter (KUSB-488, Keithley) and a GPIB cable, and the entire testing process was run automatically with a computer-controlled program. The reverse current method was used in the Hall effect measurements to avoid the thermoelectric offset current. A 9060-type electromagnet provided the magnetic field, and a gauss meter (420, Lakeshore) was used to calibrate the magnetic field. The strength of the magnetic field was 1.0 T. For the temperature-dependent *ρ* measurements, liquid nitrogen was used to obtain low temperatures of 95 to 275 K. The temperature was measured by connecting a standard K-type thermocouple to the voltage meter, with a 0 °C ice-water mixture used as the cold end. As diamond exhibits good thermal conductivity, the thermocouple was connected to the bare diamond in the DAC to ensure accurate measurements.

The first-principles calculations were performed using density functional theory and the pseudopotential method, and CASTEP code was used for calculating the electric structure. The exchange and correlation terms were described using a generalized gradient approximation (GGA) in the Perdew-Burke-Ernzerhof (PBZ) scheme. Structural optimizations were performed using the Broyden-Fletcher-Goldfarb-Shanno minimization algorithm provided in this code. Integration in the Brillouin zone was performed using special k points generated by 7 × 7 × 7 and 10 × 7 × 3 mesh parameter grids for the *F-43m* and *Imma* phases, respectively. The one-electron valence state was expanded on the basis of a plane wave with the cutoff energies of 528 eV and 580 eV, respectively. The structure parameters obtained from previous study after optimized^16, 29^.
